# Carcinoembryonic antigen as a marker of radioresistance in colorectal cancer: a potential role of macrophages

**DOI:** 10.1186/s12885-018-4254-4

**Published:** 2018-03-27

**Authors:** Eng-Yen Huang, Jen-Chieh Chang, Hong-Hwa Chen, Chieh-Ying Hsu, Hsuan-Chih Hsu, Keng-Liang Wu

**Affiliations:** 1 Department of Radiation Oncology, Kaohsiung Chang Gung Memorial Hospital, Chang Gung University College of Medicine, Kaohsiung, Taiwan; 2 School of Traditional Chinese Medicine, Chang Gung University College of Medicine, Kaohsiung, Taiwan; 3 Department of Medical Research, Kaohsiung Chang Gung Memorial Hospital, Chang Gung University College of Medicine, Kaohsiung, Taiwan; 4 Division of Colonic and Rectal Surgery, Department of Surgery, Kaohsiung Chang Gung Memorial Hospital, Chang Gung University College of Medicine, Kaohsiung, Taiwan; 5 Division of Hepatogastroenterology, Department of Internal Medicine, Kaohsiung Chang Gung Memorial Hospital, Chang Gung University College of Medicine, Kaohsiung, Taiwan

**Keywords:** Rectal cancer, Carcinoembryonic antigen, Radiotherapy, Macrophage, Radioresistance

## Abstract

**Background:**

We sought to identify the carcinoembryonic antigen (CEA) as a marker of radioresistance in rectal cancer.

**Methods:**

From July 1997 to January 2008, 104 patients with stage II or III rectal cancer who were treated with post-operative radiotherapy (PORT) were included in this study. The doses of radiotherapy ranged from 45 to 54.6 Gy. The CEA levels were measured before surgery. We analyzed the actuarial rates of overall survival (OS), distant metastasis (DM), and local recurrence (LR) using Kaplan-Meier curves. Multivariate analyses were performed with Cox regression models. We used THP-1 monocyte cell lines for macrophage differentiation (M0, M1 or M2). The RNA extracted from the macrophages was analyzed via a genomic method in the core laboratory. The radiosensitivities of CEA-rich LS1034 cells were compared between cells with and without the conditioned media from CEA-stimulated macrophages.

**Results:**

Preoperative CEA levels ≥10 ng/mL were independent predictive factors for OS (*p* = 0.005), DM (*p* = 0.026), and LR (*p* = 0.004). The OS rates among the patients with pretreatment CEA levels < 10 ng/mL and ≥10 ng/mL were 64.5% and 35.9% (*p* = 0.004), respectively. The corresponding rates of DM were 40.6% and 73.1% (*p* = 0.024). The corresponding rates of LR were 6.6% and 33.9% (*p* = 0.002). In the M0 macrophages, exogenous CEA elicited a dose-response relationship with M2 differentiation. In the CEA-stimulated M0 cells, some mRNAs were upregulated by as much as 5-fold, including MMP12, GDF15, and JAG1. In the CEA-stimulated M2 cells, a 4-fold up-regulation of GADD45G mRNA was noted. The conditioned media from the CEA-stimulated M2 cells elicited an increase in the numbers of LS180, SW620, and LS1034 cells after irradiation. CEA caused the M2 differentiation of the macrophages.

**Conclusion:**

Pretreatment CEA levels ≥10 ng/mL are a significant risk factor for OS, DM, and LR following PORT for rectal cancer. CEA causes radioresistance in the presence of M2 macrophages. More comprehensive examinations prior to surgery and intensive adjuvant therapy are suggested for patients with CEA levels ≥10 ng/mL. Further studies of these mechanisms are needed.

## Background

Radiotherapy plays a major role in the management of many types of cancer. There are challenges regarding why tumors are resistant to radiation. Although preoperative concurrent chemoradiotherapy (CCRT) or short-course radiotherapy [1] are the main treatments for rectal cancer, some patients choose surgery as their initial therapy for personal reasons. However, the influence of the tumor marker carcinoembryonic antigen (CEA) on the prognoses is not clear in patients who undergo post-operative radiotherapy (PORT). Our prior study first noted that pretreatment serum CEA levels are associated with local recurrence (LR) after radiotherapy for cervical cancer [2]. Therefore, we were interested in the role of CEA in a different cancer. Rectal cancer was a good candidate because radiotherapy is usually applied, and elevated serum CEA levels are noted in rectal cancer patients.

CEA is a complex glycoprotein with different glycosylations that cause different molecular weights between normal and cancer cells. The activation of glycosylphosphatidylinositol phospholipase D can release membrane-bound CEA and result in shedding [3]. Genetic manipulation of CEA in cancer cell lines promotes CEA release from cell membrane [4–6]. Upon its secretion, CEA promotes colorectal cancer cell metastasis especially to the liver. Kupffer cells are specialized macrophages and enhance liver metastasis [7] through their CEA receptors [8]. Tumor-associated macrophages (TAMs) are heterogeneous in human malignant tumors. TAMs are nearly always associated with poor prognoses. However, the TAMs of colorectal cancer are correlated with favorable prognoses [9]. Based on the literature and our previous studies, we hypothesized that cancer cells secrete CEA to modulate macrophage differentiation and subsequently increase the radioresistance of cancer cells. The specific aims of this study were to examine whether CEA enhances radioresistance in patients with rectal cancer and assess the involvement of CEA-stimulated macrophages in radioresistance.

## Methods

### Patient collection

Patients with histologically proven pT3–4 or N1–2 adenocarcinomas of the rectum who were treated with curative-intent surgery and post-operative radiotherapy were reviewed in this study. Patients who did not receive the planned dose (45–55 Gy) of radiotherapy or exhibited distant metastasis were excluded from the study. Between July 1997 and January 2008, 104 patients were included in the study. The characteristics of patients are presented in Table 1. This study was approved by the Institutional Review Board of our hospital (99-3189B).

**Table 1:** Patient characteristics (*n* = 104)

Parameters	No. (%) or Mean ± SEM
Age (years)	55.9 ± 11.5
Stage	
II	20 (19.2%)
III	84 (80.8%)
T stage	
T2	4 (3.8%)
T3	53 (51.0%)
T4	47(45.2%)
N stage	36 (19.1%)
N0	20 (19.2%)
N1	59 (56.7%)
N2	25 (24.0%)
APR	
No	61 (58.7%)
Yes	43 (41.3%)
Anal verge (cm)	
≤5	59 (56.7%)
6–10	34 (32.7%)
> 10	11 (10.6%)
Pretreatment CEA level (ng/mL)	17.3 ± 3.7
< 5	56 (58.3%)
5–10	18 (17.3%)
≥ 10	30 (28.8%)
EBRT dose (Gy)	
45–50.4	6 (5.8%)
52.2–54.6	98 (94.2%)

### Radiation therapy

In general, the upper margin of the pelvic irradiation was delivered with 10- or 15-MV photons through 3-dimensional conformal radiotherapy (3D-CRT) or intensity-modulated radiotherapy (IMRT) techniques. Typically, the upper margin was 1.5 cm above the level of the sacral promontory. The posterior margin was 1.5–2 cm behind the anterior bony sacral margin. The dose was 45–55 Gy in 25 to 28 fractions that were administered within 5–6 weeks. Typically, the patients received 5-fluorouracil (5-FU)-based chemotherapy concurrently with the radiotherapy.

### Follow-up and statistics

After the completion of the radiotherapy, follow-ups of the patients were performed and included physical examinations, laboratory tests, chest X-rays, colonoscopies, and computed tomography (CT) scans. Local recurrence (LR) and distant metastasis (DM) were confirmed by biopsy, physical examination, or imaging diagnosis. The overall survival (OS), LR, and DM rates were calculated via the Kaplan-Meier method, and the differences were examined with the log rank test. The interval to the last follow-up was defined by the last administration of radiotherapy. Multivariate analysis was performed using the Cox proportional hazard model with a forward stepwise procedure for OS, LR, and DM. The relative risks of these variables are represented by the hazard ratios (HRs) with the 95% confidence intervals (CIs). All variables, including age (< 57 and ≥57 years), stage, CEA level (< 10 and ≥10 ng/mL), tumor location, and operation, were treated as categorical data. The statistics were performed on a personal computer using the SPSS 17.0 software (SPSS Inc., Chicago, IL) for MS Windows®.

### Cell culture

THP-1 (TIB-202), SW48 (CCL-231), SW620 (CCL-227), DLD-1 (CCL-221), LS1034 (CRL-2158), HCT116 (CCL-247), LS174T (CL-188), LS180 (CL-187), and Caco2 (HTB-37) cells were purchased from the American Type Culture Collection. The cells were grown in the suggested media with 4.5 g/l glucose and supplemented with 10% fetal bovine serum (Gibco Life Technologies, Grand Island, NY, USA) and antibiotics at 37°C in 5% CO_2_. The media were changed every 3 days.

### Macrophage differentiation

Harvested differentiated THP-1 cells were seeded at a density of 2×10^6^ cells in 25 T flasks. The THP-1 cells were differentiated to the attached state (M0) with 12-myristate 13-acetate (PMA; Sigma) at 50 ng/ml for 6 h. The attached cells generated M2-polarized macrophages following treatment with PMA plus 20 ng/ml IL-4 and 20 ng/ml IL-13 (R&D Systems) for another 66 h. The M1 polarization medium was treated with PMA plus 10 ng/ml LPS (Sigma) and 20 ng/ml IFN-γ (R&D systems) for 66 h.

### Flow cytometry analysis

Differentiated THP-1 cells were washed with PBS three times. To detect M1 markers, the cells were incubated with CCR7-APC (MACS) monoclonal antibody for 1 h. To detect the M2 markers, the cells were incubated with primary CD163 (Santa Cruz) monoclonal antibody for 1 h. After 1 h, the cells were washed with PBS and then incubated with FITC-conjugated goat anti-mouse secondary antibody for 1 h. The samples were analyzed by flow cytometry on an FLSR II flow cytometry system (BD).

### Radiosensitivity assay

In addition to IL-4 and IL-13, human CEA (300 ng/mL) (Abcam) was also added for 66 h during M2 differentiation. We collected CEA-stimulated M2 conditioned medium (CM) for 1: 1 mixture of culture of colon cancer cells. After 24 h, cancer cells were irradiated with 8 Gy then seeded in 96 well plate for 72 h. The cells were counted using a Cell Proliferation Reagent WST-1 (Roche Molecular Biochemicals, Mannheim, Germany). The analysis method was applied according to the instructions supplied by the manufacturer. Briefly, WST-1 (20μl) was added to well for 30 min the OD (450 nm) was measured for counting of cell number. No CEA-stimulated M2 CM, CEA + cancer cell-free medium, and cancer cell-free medium were also added to culture medium of colon cancer cells for comparison. OD after irradiation was compared between M2 CM and M2 CM + CEA group using paired-t test. The corresponding OD was also compared between cancer cell-free medium and cancer cell-free medium +CEA group.

### Gene expression profiling

The total RNAs were run on an Agilent RNA 6000 Nanochip (Agilent Technologies, Palo Alto, CA) using an Agilent 2100 Bioanalyzer (Agilent Technologies, Palo Alto, CA) to detect the RNA quality and to determine the concentration of RNA using a NanoDrop spectrophotometer (Thermo Science, Wilmington, DE). Only samples with A260/A280 ratios of 1.9 to 2.1 and RNA integrity numbers (RINs) over 7.0 were used for the subsequent cRNA amplification analysis. A total of 500 ng of RNA was used for the synthesis of the first strand cDNA and the in vitro transcription (IVT) of the cRNA using an Illumina TotalPrep RNA Amplification Kit (Ambion, Austin, TX) according to the manufacturer’s directions. Briefly, a single 20-μl aliquot of RNA and reverse transcription master mix was incubated for 2 h at 42°C to synthesize the first strand cDNA. Eighty microliters of second strand master mix was added, and the solution was incubated for 2 h at 16°C to synthesize the second strand cDNA. After incubation with 7.5 μl IVT master mix for 14 h at 37°C and purification, the size distribution of the synthesized biotin-labeled cRNA was evaluated with an Agilent 2100 Bioanalyzer. Gene expression was analyzed using the Sentrix HumanHT-12 v4 Expression BeadChip (Illumina, San Diego, CA) to generate the expression profiles of more than 47,000 probes with 750 ng of labeled cRNA for each sample according to the manufacturer’s protocol. After hybridization for 20 h at 58°C, washing and fixation, the HumanHT-12 Expression BeadChip was detected with Cy3-streptavidin (GE Healthcare, Little Chalfont, Buckinghamshire, UK) and quantitated using an Illumina BeadStation 500GX (Illumina, San Diego, CA). The expression intensity measures were obtained and extracted with the gene expression module version 1.9.0 of the Illumina GenomeStudio V2011.1 software (Illumina, San Diego, CA) for further bioinformatics analysis.

### Microarray data analysis

All of the gene expression data were log2 transformed and exported using the Partek Report Plugin 2.16 of the Illumina GenomeStudio platform in the Partek Genomics Suite version 6.6. The gene expression levels in the samples were quantile-normalized in the Partek analysis software and subsequently analyzed for differential genetic expression profiles. The analysis of variance (ANOVA) algorithm of the Partek software was used to compare the differentially expressed genes between the CEA-stimulated and vehicle-stimulated macrophages. Changes in gene expression levels of 2- to 5-fold in the CEA-stimulated samples relative to the vehicle-stimulated controls with *p* values < 0.05 were considered to be significant. Visualizations of heatmaps of the hierarchical clusterings and the biological interpretations from Gene Ontology (GO) Enrichment and the KEGG pathway are presented for the significant genes.

## Results

### Treatment outcomes

The median follow-up time for the patients who are alive was 98.9 months (range 45–156 months). The 5-year OS, DM, and LR values were 56.2%, 49.8%, and 14.1%, respectively. DM was noted in 50 patients. Among the 38 patients who underwent CEA examinations upon DM, 26 patients (68.4%) had CEA levels > 5 ng/mL. The 5 most common sites of DM were the lungs (16.8%), liver (14.0%), para-aortic lymph node (8.4%), carcinomatosis (6.6%), and brain (5.6%), which were noted in 18, 15, 9, 7, and 6 patients, respectively. LR was noted in 13 patients. Among 5 patients who underwent CEA examinations upon LR, 4 patients (80%) had CEA levels > 5 ng/mL. The DM rates were 92.3% and 41.8% in the patients with and without LR (*p* = 0.001), respectively. The median time to DM was 16.4 months (range 1–86). The median time to LR was 13.7 months (range 4–87).

**Fig. 1:**
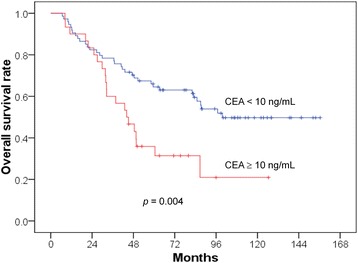
Lower overall survival rate in the patients with CEA levels ^3^ 10 ng/mL

**Fig. 2:**
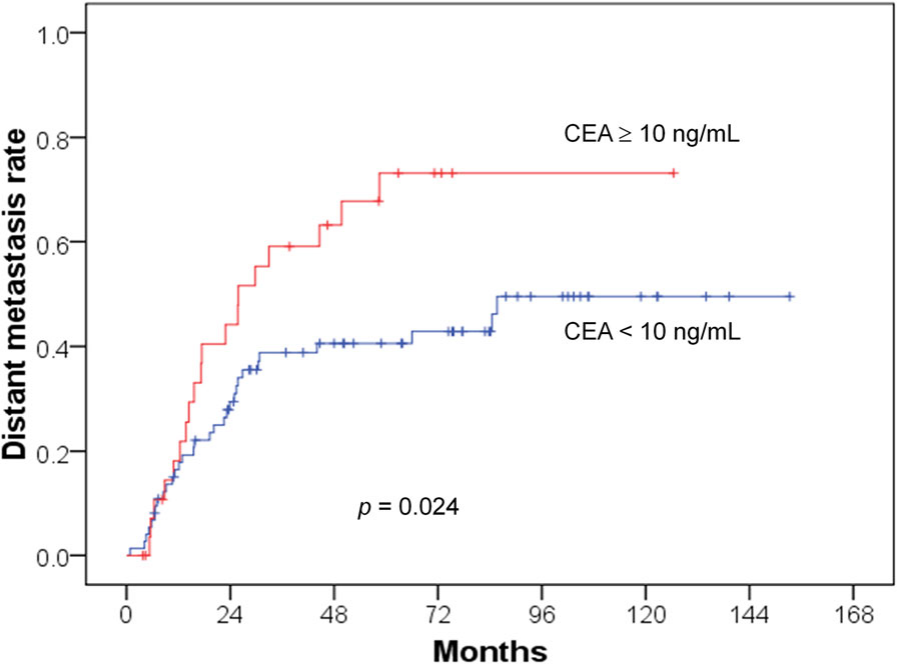
Higher distant metastasis rate in the patients with CEA levels ^3^ 10 ng/mL

**Fig. 3:**
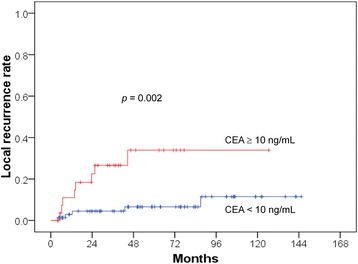
Higher local recurrence rate in the patients with CEA levels ^3^ 10 ng/mL

The univariate and multivariate analyses are presented in Tables 2, 3 and 4. The 5-year OS rates were 64.5% and 35.9% (*p* = 0.004) in the patients with pretreatment CEA levels of < 10 and ≥ 10 ng/mL (Fig. 1), respectively. The DM rates were 40.6% and 73.1% (*p* = 0.024) in the patients with pretreatment CEA levels of < 10 and ≥ 10 ng/mL (Fig. 2), respectively. The LR rates were 6.6% and 33.9% (*p* = 0.002) in the patients with pretreatment CEA levels of < 10 and ≥ 10 ng/mL (Fig. 3), respectively. The multivariate analyses revealed that pretreatment CEA levels of < 10 and ≥ 10 ng/mL were the only independent predictive factors for OS (*p* = 0.005; HR: 2.229; 95% CI 1.272–3.906), DM (*p* = 0.026; HR: 1.923; 95% CI 1.080–3.423), and LR (*p* = 0.004; HR: 5.340; 95% CI 1.682–16.955).

**Table 2:** Univariate and multivariate analysis of overall survival (OS)

Parameters	5-year OS	*p* value	Hazard ratio (95% CI)	*p* value
Age		0.759		0.513
< 57	56.3%		reference	
≥ 57	56.2%		—	
Stage		0.434		0.513
II	62.9%		reference	
III	54.5%		—	
T stage		0.121		0.204
T2	100%		reference	
T3	60.2%		—	
T4	47.5%		—	
N stage		0.736		0.798
0	62.9%		reference	
1	55.7%		—	
2	52.0%		—	
APR		0.431		0.459
No	56.7%		reference	
Yes	55.7%		—	
Anal verge (cm)		0.196		0.169
≤5	55.9%		reference	
6–10	63.5%		—	
> 10	36.4%		—	
Pretreatment CEA level		0.004		0.005
< 10 ng/mL	64.5%		reference	
≥ 10 ng/mL	35.9%		2.229 (1.272–3.906)	

*Abbreviations*: CI = confidence interval; CEA = Carcinoembryonic antigen

**Table 3:** Univariate and multivariate analysis of distant metastasis (DM)

Parameters	5-year DM	*p* value	Hazard ratio (95% CI)	*p* value
Age		0.459		0.694
< 57	53.2%		reference	
≥ 57	46.2%		—	
Stage		0.065		0.100
II	35.5%		reference	
III	52.8%		—	
T stage		0.200		0.286
T2	0%		reference	
T3	44.6%		—	
T4	59.3%		—	
N stage		0.086		0.149
0	35.5%		reference	
1	49.0%		—	
2	62.0%		—	
APR		0.643		0.667
No	47.3%		reference	
Yes	53.7%		—	
Anal verge (cm)		0.071		0.066
≤5	53.3%		reference	
6–10	33.0%		—	
> 10	84.1%		—	
Pretreatment CEA level		0.024		0.026
< 10 ng/mL	40.6%		reference	
≥ 10 ng/mL	73.1%		1.923 (1.080–3.423)

**Table 4:** Univariate and multivariate analysis of local recurrence (LR)

Parameters	5-year LR	*p* value	Hazard ratio (95% CI)	*p* value
Age		0.412		0.755
< 57	18.8%		reference	
≥ 57	9.0%		—	
Stage		0.723		0.998
II	14.1%		reference	
III	14.1%		—	
T stage		0.584		0.806
T2	0%		reference	
T3	12.2%		—	
T4	17.7%		—	
N stage		0.917		0.898
0	14.1%		reference	
1	13.5%		—	
2	14.9%		—	
APR		0.121		0.134
No	9.9%		reference	
Yes	20.2%		—	
Anal verge (cm)		0.523		0.341
≤5	14.7%		reference	
6–10	11.1%		—	
> 10	20.5%		—	
Pretreatment CEA level		0.002		0.004
< 10 ng/mL	6.6%		reference	
≥ 10 ng/mL	33.9%		5.340 (1.682–16.955)

### CEA stimulated M0 cells toward M2 differentiation

**Fig. 4:**
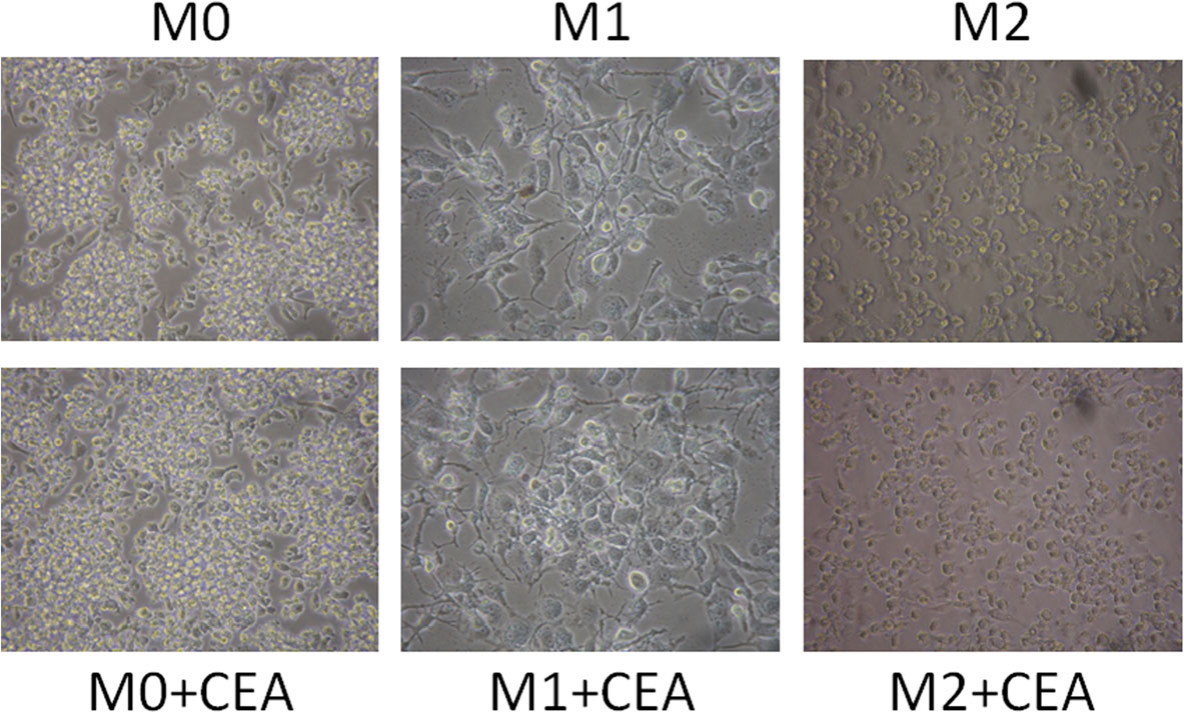
Morphologies of the M0, M1 and M2 macrophages with and without CEA stimulation

**Fig. 5:**
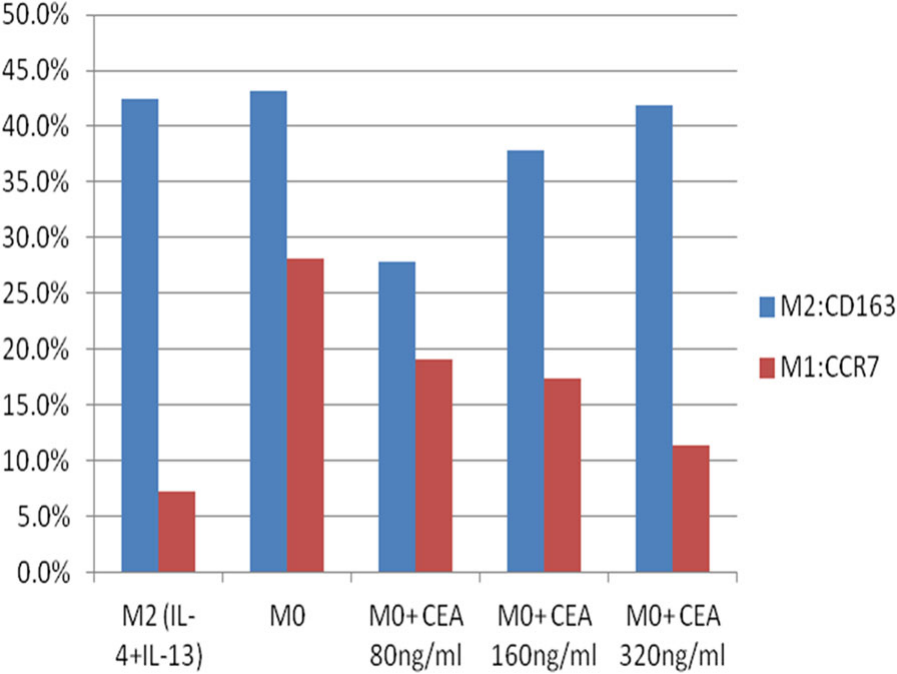
M1 (CCR7) and M2 (CD163) expression after CEA stimulation of M0 cells

Different morphologies were noted between the M0, M1, and M2 cells (Fig. 4). We used CCR7 and CD163 as markers of M1 and M2 cells, respectively. We noted a dose-response relationship of CEA with M2 differentiation (Fig. 5). We tested the CEA levels of the supernatants from different colon cancer cell lines (SW48, SW620, DLD-1, HCT116, LS1034, LS174T, and Caco2). We noted 18.392 ng/mL in LS1034 cells and 8.357 ng/mL in LS174T. Levels lower than 1 ng/mL were noted in the other cell lines (Table 5). Hence, we used the LS1034 cells for radiosensitivity testing.

**Table 5:** CEA concentration in supernatant of different colon cancer cell lines

Cells	CEA (ng/ml)	medium	CEA (ng/ml)
SW48	0.367	L-15	0.087
SW620	0.266	L-15	0.087
DLD-1	0.557	RPMI	0.51
LS1034	18.392	RPMI	0.51
HCT116	0.379	5A	0.357
LS174T	8.357	MEM	0.334
caco2	0.692	DMEM	0.332

Determined by radioimmunoassay (RIA)

### Gene expression in CEA-stimulated macrophages

**Fig. 6:**
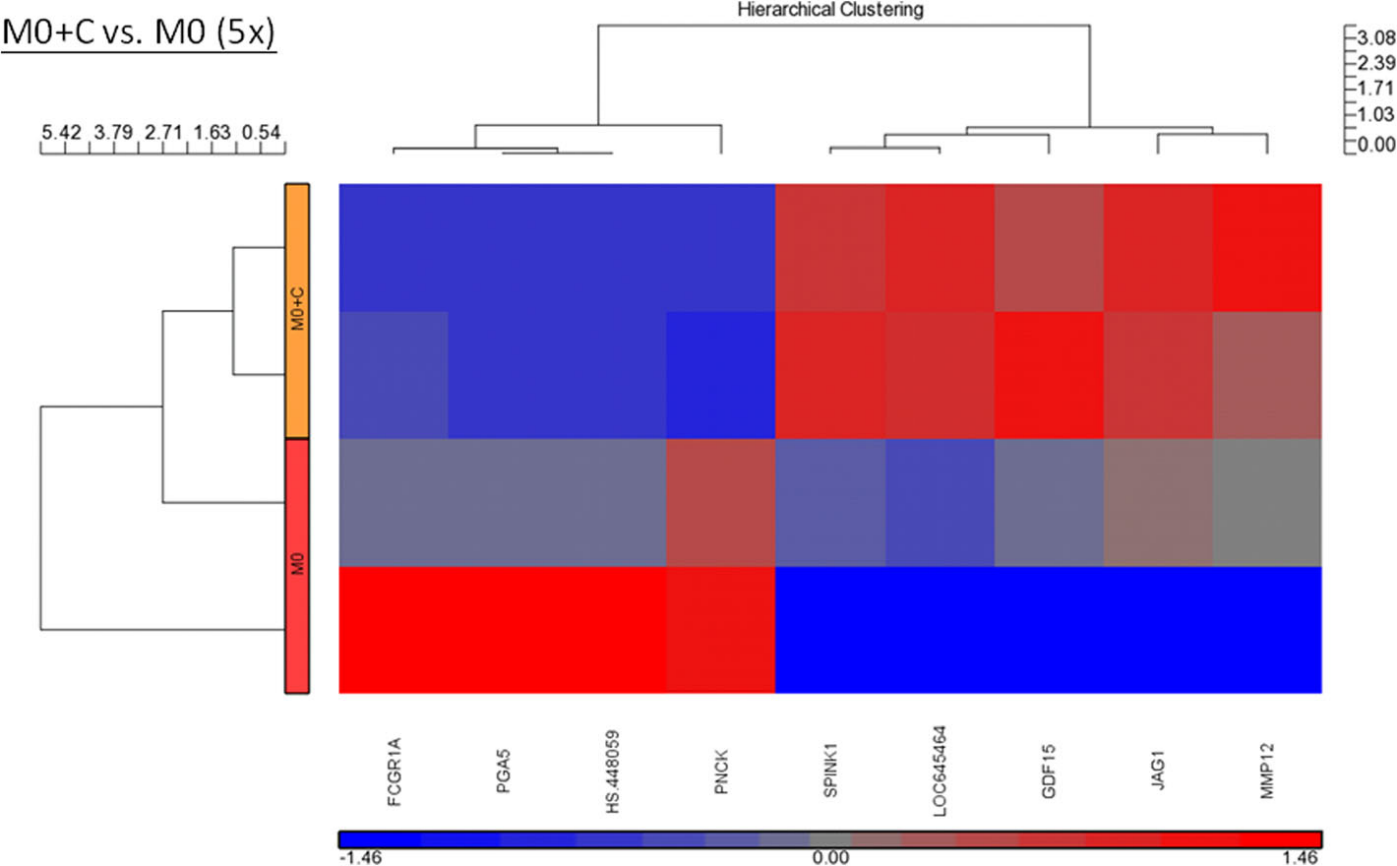
Hierarchical clustering of the up- and down-regulated genes (5-fold) after CEA (2 μg/mL) stimulation of M0 macrophages

**Fig. 7:**
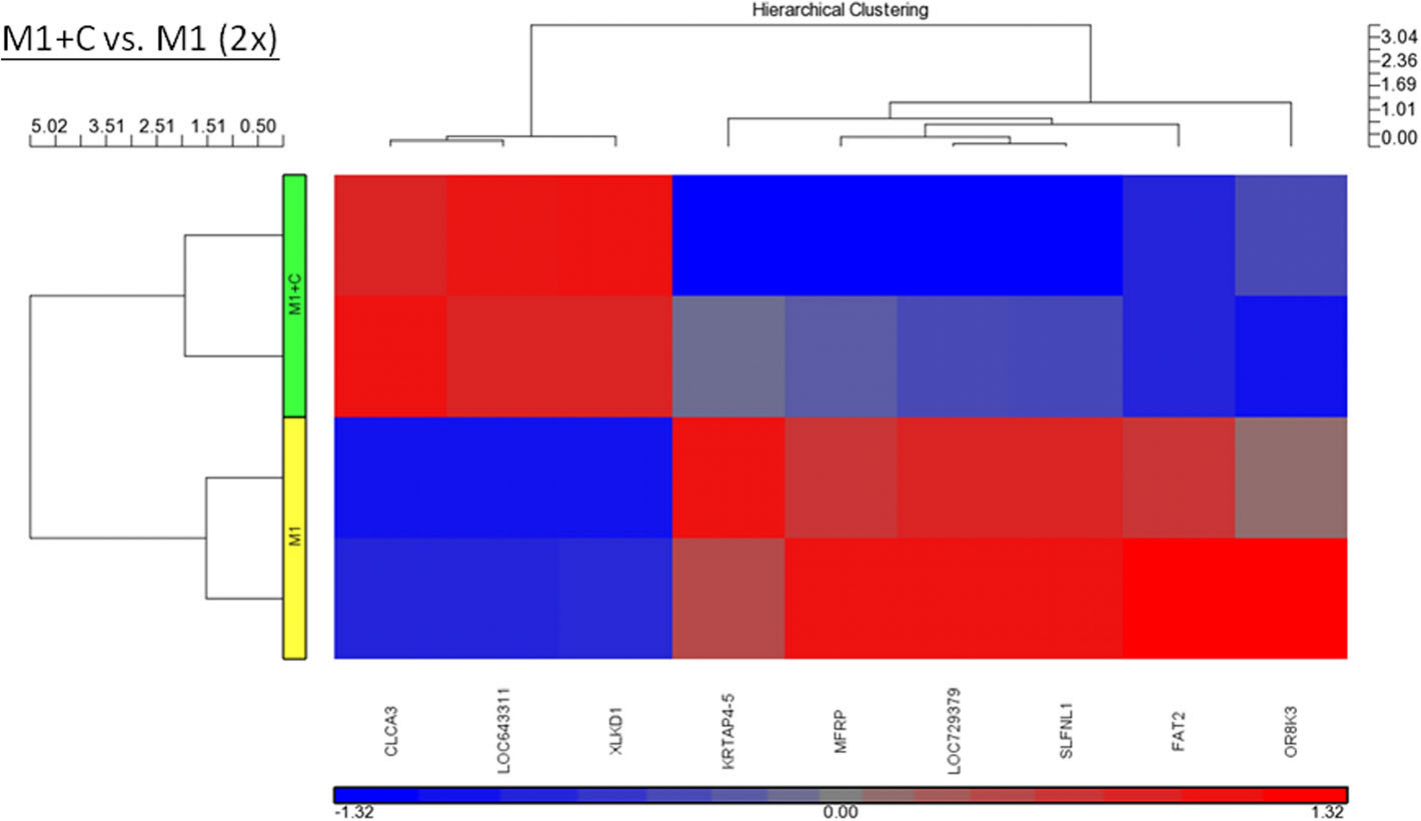
Hierarchical clustering of the up- and down-regulated genes (2-fold) after CEA (2 μg/mL) stimulation of M1 macrophages

In the CEA-stimulated M0 macrophages (Fig. 6 and Table 6), four mRNAs that exhibited at least 5-fold down-regulation compared with the vehicle-stimulated M0 cells were noted. Five mRNAs, including MMP12, GDF15, and JAG1, exhibited at least 5-fold up-regulation. In the CEA-stimulated M1 macrophages (Fig. 7 and Table 7), three mRNAs were at least 2-fold down-regulated compared with the vehicle-stimulated M1 cells. Six mRNAs exhibited at least 2-fold up-regulation. In the CEA-stimulated M2 macrophages, 15 mRNAs exhibited at least 2-fold down-regulation compared with the non-stimulated M2 cells. Twenty-four mRNAs exhibited at least 2-fold up-regulation. The GADD45G mRNA achieved a 4-fold up-regulation of expression. GADD45G is a stress response gene to radiotherapy.

**Table 6:** Genes of up and down regulation (5-fold) after CEA stimulation in M0 macrophage

Symbol	*p*-value (M0 + CEA vs. M0)	Log fold (M0 + CEA vs. M0)	Fold-Change (M0 + CEA vs. M0)
MMP12	0.0257923	0.701613	5.03052
FCGR1A	0.0422142	−0.70421	−5.06069
PNCK	0.0398482	−0.70525	−5.07281
PGA5	0.0297503	−0.7423	−5.52462
HGC6.3	0.0373702	−0.78718	−6.12611
GDF15	0.00840181	0.785626	6.10416
JAG1	0.0437505	0.720147	5.24985
SPINK1	0.00346519	1.14264	13.888
LOC645464^a^	0.0130519	0.739874	5.49381

MMP12 = Matrix metalloproteinase-12

FCGR1A = High affinity immunoglobulin gamma Fc receptor I

PNCK = Pregnancy Up-Regulated Nonubiquitous CaM Kinase

*PGA5 = pepsinogen 5*

HGC6.3 = Human hypothetical LOC100128124

GDF15 = Growth/differentiation factor 15

JAG1 = Jagged1

SPINK1 = serine protease inhibitor Kazal-type 1

^a^This record has been withdrawn by NCBI because the model on which it was based was not predicted in a later annotation

**Table 7:** Genes of up and down regulation (2-fold) after CEA stimulation in M1 macrophage

Symbol	*p*-value (M1 + CEA vs. M0)	Log fold (M1 + CEA vs. M0)	Fold-change (M1 + CEA vs. M0)
KRTAP4–5	0.0159843	−0.30188	−2.00392
SLFNL1	0.0316286	−0.35666	−2.27333
LOC729379^a^	0.000549518	−0.3691	−2.3394
CT47B1	0.000259443	0.439442	2.75069
XLKD1	0.00563431	0.331138	2.14357
FAT2	0.00944461	−0.3035	−2.01141
CLCA3	0.00767531	0.341114	2.19338
MFRP	0.00689281	−0.40706	−2.55305
OR8K3	0.0121585	−0.39214	−2.46683

KRTAP4–5 = keratin associated protein 4–5

SLFNL1 = schlafen like 1

LOC729379

CT47B1 = Cancer/Testis Antigen Family 47, Member B1

XLKD1 = extracellular link domain containing 1

FAT2 = FAT atypical cadherin 2

CLCA3 = Chloride channel accessory 3,

MFRP = membrane frizzled-related protein

OR8K3 = olfactory receptor family 8 subfamily K member 3

^a^This record has been withdrawn by NCBI because the model on which it was based was not predicted in a later annotation

### The conditioned media from CEA-stimulated M2 macrophages enhanced radioresistance

**Fig. 8:**
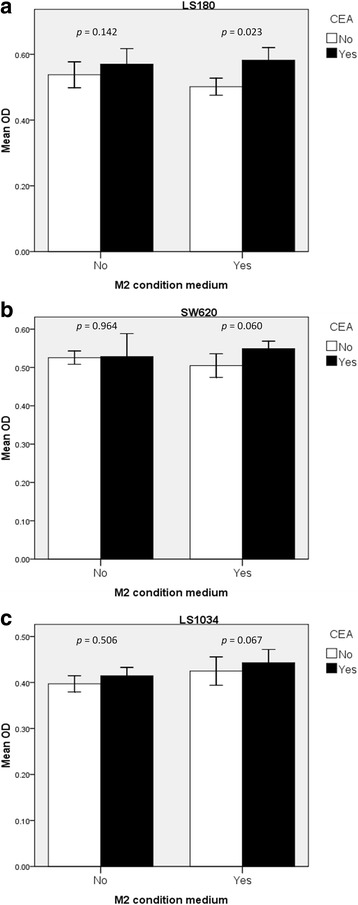
CEA (320 ng/mL) enhanced the radioresistance of (**a**) LS180, (**b**) SW620, and (**c**) LS1034 cells cultured with M2 conditioned medium. However, CEA did not change radiosensitivity in cancer cells without M2 conditioned medium. Each experimental group included triplicated studies

The conditioned media from M2 macrophages or medium only was applied to the LS1034, SW620, LS180 cells prior to irradiation. We compared the OD for cell proliferation between the CEA-stimulated and vehicle-stimulated cells after irradiation. We found a higher OD in the CEA-stimulated group with M2 conditioned medium but not medium only group (Fig. 8).

## Discussion

Regardless of an early report of preoperative CEA in recurrent colorectal cancer [10], the role of CEA in the prognoses may be dependent on the disease site (i.e., colon or rectal cancer), whether radiotherapy is applied, the end points examined (i.e., OS, DM, and LR), and the time at which the CEA level is measured. Therefore, CEA is involved in cancer progression and recurrence (following surgery or radiotherapy) in some studies, especially in the era of the established roles of neoadjuvant and adjuvant radiotherapies. In rectal cancer, the role of the pretreatment CEA level has been well studied in terms of neoadjuvant therapy with the endpoints of pathologically complete responses [11–17] and OS [18]. Normalization of the CEA level after neoadjuvant therapy is also a significant factor in pathologically complete responses [19] and prognoses [14, 20, 21].

There is only one study that has examined the prognostic role of preoperative CEA levels in Dukes’ B and C patients who have undergone PORT for rectal cancer [22]. The authors of this study found no significant role of CEA in LR. A second study [23] included stage II and III preoperative and post-operative CCRT patients. The 5-year OS, DM, and LR rates were 49.4%, 14.9%, and 66.0%, respectively. These authors noted that a CEA level > 3 ng/mL affected the OS and LR but not the DM based on univariate analysis. The CEA level was treated as a continuous variable in a multivariate analysis and was found to be an independent factor for OS.

In the present study, we included only PORT and not neoadjuvant patients. We studied not only the OS but also the LR and DM and noted significant roles of CEA in all of the end points. The preoperative CEA level may serve as a marker of occult metastasis and radioresistance to PORT in rectal cancer. This study also confirmed the results of our previous study that investigated the role of pretreatment CEA levels in the LRs, DMs, and OSs of patients undergoing definitive radiotherapy for cervical cancer [2, 24]. We also found that LR usually preceded DM, and a higher DM rate was noted in patients with LR than in those without LR. We observed an early separation of the Kaplan-Meier curves for LR (at approximately < 6 months; Fig. 3) between the patients with high and low CEA levels but did not observe this early separation for OS (the separation occurred at approximately 2 years; Fig. 1) or DM (the separation occurred at approximately 1 year; Fig. 2). CEA first involves LR, which increases the DM and thus affects survival. Therefore, the reviewed literature and our results support the role of CEA in LR. In other words, CEA may be a marker of radioresistance. For this reason, we attempted to investigate the mechanism by which CEA affects radioresistance in an in vitro study.

Macrophages are the major immune cells in cancer microenvironments [25]. Monocytes in cancer tissues can differentiate into M0 macrophages. If M0 cells are stimulated with IFN-γ, LPS, and TNF-α, they differentiate into M1 macrophages that exhibit pro-inflammatory functions. If M0 cells are stimulated with IL-4, IL-10, and the Toll-like receptor, they differentiate into M2 macrophages that exhibit anti-inflammatory functions that improve wound-healing, angiogenesis, and remodeling. However, cancer cells can co-operate with M2 macrophages to promote proliferation, migration, invasion, angiogenesis and metastasis. For example, M2 macrophages secrete EGF, which promotes cancer cell progression [26].

The interaction of CEA and macrophages has been noted in some studies [7, 8]. Cui et al. noted a positive correlation of M2 cells but not M1 cells with the preoperative CEA level in colorectal cancer patients with liver metastases [27]. Therefore, we suggest that CEA can stimulate macrophages toward M2 differentiation and subsequently release radioresistant molecules that affect cancer cells. The interesting finding of the present study is that CEA stimulates GADD45G mRNA expression by M2 macrophages. GADD45G is a DNA damage response gene [28] that is associated with radiation. GADD45G also induces hematopoietic stem cell differentiation [29]. Furthermore, GADD45G increases TNF-α and IL-6 production in LPS-stimulated THP-1 cells [30].

We noted the expressions of some genes in the CEA-stimulated M0 macrophages. Some of these genes may be involved in macrophage-mediated cancer progression. MMP12 can enhance the progression of nasopharyngeal carcinomas through the heterogeneous nuclear ribonucleoprotein (hnRNP) K [31]. Interestingly, CEA increases IL-6 and TNF-α expressions through hnRNP M4 [8]. Additional mechanisms are worth investigating. Growth differentiation factor 15 (GDF15) is a superfamily of TGF-β that is expressed by M2 macrophages. Human jagged 1 (JAG1) is a receptor of the Notch-1 ligand, and JAG1 expression is under the control of hematopoietic growth factors [32]. In the present study, CEA was found to increase JAG1 expression. Because JAG1 and Notch-1 are poor prognostic factors in several cancers [33–35], it is reasonable to propose that the role of CEA in cancer progression is mediated through JAG1 and Notch-1 expressions in M0 cells.

There are some limitations of this study. A high DM rate was noted in the present study. An inadequate number of lymph node examinations was noted in our hospital, and this likely affected the prognoses [36]. Hence, the stages of the patients included in the present study might have been underestimated, and occult metastases might have appeared. These occult metastases resulted in the DM rate. The sample size was limited because most of the patients referred to our department underwent preoperative radiotherapy, and not all pT3–4 and pN1–2 patients were referred for PORT. The data presented is sufficient to generate the hypothesis rather than being robust enough to prove the point. Other causes for radioresistance are not accounted for. More direct studies of the stromal cells are required to prove the issue.

## Conclusion

Based on our results and the literature, CEA may stimulate M2 but not M1 differentiation. CEA causes radioresistance in the presence of M2 macrophages. Pretreatment CEA levels ≥ 10 ng/mL are a significant risk factor for OS, DM, and LR following PORT for rectal cancer. More comprehensive examinations prior to surgery and intensive adjuvant therapy are suggested for patients with CEA levels ≥10 ng/mL. Further translational studies of these mechanisms are needed.

## Abbreviations

CCRT: Concurrent chemoradiotherapy
CEA: Carcinoembryonic antigen
CI: Confidence interval
DM: Distant metastasis
HR: Hazard ratio
LR: Local recurrence
OS: Overall survival
PORT: Post-operative radiotherapy

## References

[1] Chen C, Sun P, Rong J, Weng HW, Dai QS, Ye S (2015). Short course radiation in the treatment of localized rectal cancer: a systematic review and meta-analysis. Sci Rep.

[2] Huang EY, Hsu HC, Sun LM, Chanchien CC, Lin H, Chen HC, Tseng CW, Ou YC, Chang HY, Fang FM, Huang YJ, Wang CY, Lu HM, Tsai CC, Ma YY, Fu HC, Wang YM, The WCJ (2011). Prognostic value of pretreatment carcinoembryonic antigen after definitive radiotherapy with or without concurrent chemotherapy for squamous cell carcinoma of the uterine cervix. Int J Radiat Oncol Biol Phys.

[3] Yamamoto Y, Hirakawa E, Mori S, Hamada Y, Kawaguchi N, Matsuura N (2005). Cleavage of carcinoembryonic antigen induces metastatic potential in colorectal carcinoma. Biochem Biophys Res Commun.

[4] Hefta LJ, Chen FS, Ronk M, Sauter SL, Sarin V, Oikawa S, Nakazato H, Hefta S, Shively JE (1992). Expression of carcinoembryonic antigen and its predicted immunoglobulin-like domains in HeLa cells for epitope analysis. Cancer Res.

[5] Terskikh A, Mach JP, Pèlegrin A (1993). Marked increase in the secretion of a fully antigenic recombinant carcinoembryonic antigen obtained by deletion of its hydrophobic tail. Mol Immunol.

[6] Naghibalhossaini F, Pakdel A, Ghaderi AA, Saberi Firoozi M (2005). Effective production of carcinoembryonic antigen by conversion of the membrane-bound into a recombinant secretory protein by site-specific mutagenesis. Pathol Oncol Res.

[7] Aarons CB, Bajenova O, Andrews C, Heydrick S, Bushell KN, Reed KL, Thomas P, Becker JM, Stucchi AF (2007). Carcinoembryonic antigen-stimulated THP-1 macrophages activate endothelial cells and increase cell-cell adhesion of colorectal cancer cells. Clin Exp Metastasis.

[8] Bajenova OV, Zimmer R, Stolper E, Salisbury-Rowswell J, Nanji A, Thomas P (2001). Heterogeneous RNA-binding protein M4 is a receptor for carcinoembryonic antigen in Kupffer cells. J Biol Chem.

[9] Komohara Y, Jinushi M, Takeya M (2014). Clinical significance of macrophage heterogeneity in human malignant tumors. Cancer Sci.

[10] Wanebo HJ, Rao B, Pinsky CM, Hoffman RG, Stearns M, Schwartz MK, Oettgen HF (1978). Preoperative carcinoembryonic antigen level as a prognostic indicator in colorectal cancer. N Engl J Med.

[11] Park YA, Sohn SK, Seong J, Baik SH, Lee KY, Kim NK, Cho CW (2006). Serum CEA as a predictor for the response to preoperative chemoradiation in rectal cancer. J Surg Oncol.

[12] Das P, Skibber JM, Rodriguez-Bigas MA, Feig BW, Chang GJ, Wolff RA, Eng C, Krishnan S, Janjan NA, Crane CH (2007). Predictors of tumor response and downstaging in patients who receive preoperative chemoradiation for rectal cancer. Cancer.

[13] Yoon SM, Kim DY, Kim TH, Jung KH, Chang HJ, Koom WS, Lim SB, Choi HS, Jeong SY, Park JG (2007). Clinical parameters predicting pathologic tumor response after preoperative chemoradiotherapy for rectal cancer. Int J Radiat Oncol Biol Phys.

[14] Park JW, Lim SB, Kim DY, Jung KH, Hong YS, Chang HJ, Choi HS, Jeong SY (2009). Carcinoembryonic antigen as a predictor of pathologic response and a prognostic factor in locally advanced rectal cancer patients treated with preoperative chemoradiotherapy and surgery. Int J Radiat Oncol Biol Phys.

[15] Restivo A, Zorcolo L, Cocco IM, Manunza R, Margiani C, Marongiu L, Casula G (2013). Elevated CEA levels and low distance of the tumor from the anal verge are predictors of incomplete response to chemoradiation in patients with rectal cancer. Ann Surg Oncol.

[16] Wallin U, Rothenberger D, Lowry A, Luepker R, Mellgren A (2013). CEA - a predictor for pathologic complete response after neoadjuvant therapy for rectal cancer. Dis Colon rectum.

[17] Probst CP, Becerra AZ, Aquina CT, Tejani MA, Hensley BJ, González MG, Noyes K, Monson JR, Fleming FJ (2016). Watch and wait?--elevated pretreatment CEA is associated with decreased pathological complete response in rectal Cancer. J Gastrointest Surg.

[18] Chung MJ, Nam TK, Jeong JU, Kim SH, Kim K, Jang HS, Jeong BK, Lee JH (2016). Can serum dynamics of carcinoembryonic antigen level during neoadjuvant chemoradiotherapy in rectal cancer predict tumor response and recurrence? A multi-institutional retrospective study. Int J Color Dis.

[19] Kleiman A, Al-Khamis A, Farsi A, Kezouh A, Vuong T, Gordon PH, Vasilevsky CA, Morin N, Faria J, Ghitulescu G, Boutros M (2015). Normalization of CEA levels post-neoadjuvant therapy is a strong predictor of pathologic complete response in rectal Cancer. J Gastrointest Surg.

[20] Yang KL, Yang SH, Liang WY, Kuo YJ, Lin JK, Lin TC, Chen WS, Jiang JK, Wang HS, Chang SC, Chu LS, Wang LW (2013). Carcinoembryonic antigen (CEA) level, CEA ratio, and treatment outcome of rectal cancer patients receiving pre-operative chemoradiation and surgery. Radiat Oncol.

[21] Ishihara S, Watanabe T, Kiyomatsu T, Yasuda K, Nagawa H (2010). Prognostic significance of response to preoperative radiotherapy, lymph node metastasis, and CEA level in patients undergoing total mesorectal excision of rectal cancer. Int J Color Dis.

[22] Bentzen SM, Balslev I, Pedersen M, Teglbjaerg PS, Hanberg-Sørensen F, Bone J, Jacobsen NO, Sell A, Overgaard J, Bertelsen K (1992). Time to loco-regional recurrence after resection of Dukes' B and C colorectal cancer with or without adjuvantpostoperative radiotherapy. A multivariate regression analysis. Br J Cancer.

[23] Weissenberger C, Von Plehn G, Otto F, Barke A, Momm F, Geissler M (2005). Adjuvant radiochemotherapy of stage II and III rectal adenocarcinoma: role of CEA and CA 19-9. Anticancer Res.

[24] Huang EY, Huang YJ, Chanchien CC (2012). Pretreatment carcinoembryonic antigen level is a risk factor for Para-aortic lymph node recurrence in addition to squamous cell carcinoma antigen following definitive concurrent chemoradiotherapy for squamous cell carcinoma of the uterine cervix. Radiat Oncol.

[25] Hao NB, Lü MH, Fan YH, Cao YL, Zhang ZR, Yang SM (2012). Macrophages in tumor microenvironments and the progression of tumors. Clin Dev Immunol.

[26] Laoui D, Movahedi K, Van Overmeire E, Van den Bossche J, Schouppe E, Mommer C, Nikolaou A, Morias Y, De Baetselier P, Van Ginderachter JA (2011). Tumor-associated macrophages in breast cancer: distinct subsets, distinct functions. Int J Dev Biol.

[27] Cui YL, Li HK, Zhou HY, Zhang T, Li Q (2013). Correlations of tumor-associated macrophage subtypes with liver metastases of colorectal cancer. Asian Pac J Cancer Prev.

[28] Smith ML, Chen IT, Zhan Q, Bae I, Chen CY, Gilmer TM, Kastan MB, O'Connor PM, Fornace AJ (1994). Interaction of the p53-regulated protein Gadd45 with proliferating cell nuclear antigen. Science.

[29] Thalheimer FB, Wingert S, De Giacomo P, Haetscher N, Rehage M, Brill B, Theis FJ, Hennighausen L, Schroeder T, Rieger MA (2014). Cytokine-regulated GADD45G induces differentiation and lineage selection in hematopoietic stem cells. Stem Cell Reports.

[30] Shin GT, Lee HJ, Kim H (2012). GADD45γ regulates TNF-α and IL-6 synthesis in THP-1 cells. Inflamm Res.

[31] Chung IC, Chen LC, Chung AK, Chao M, Huang HY, Hsueh C (2014). Matrix metalloproteinase 12 is induced by heterogeneous nuclear ribonucleoprotein K and promotes migration and invasion in nasopharyngeal carcinoma. BMC Cancer.

[32] Nomaguchi K, Suzu S, Yamada M, Hayasawa H, Motoyoshi K (2001). Expression of Jagged1 gene in macrophages and its regulation by hematopoietic growth factors. Exp Hematol.

[33] Pannequin J, Bonnans C, Delaunay N, Ryan J, Bourgaux JF, Joubert D, Hollande F (2009). The wnt target jagged-1 mediates the activation of notch signaling by progastrin in human colorectalcancer cells. Cancer Res.

[34] Lin JT, Chen MK, Yeh KT, Chang CS, Chang TH, Lin CY (2010). Association of high levels of Jagged-1 and Notch-1 expression with poor prognosis in head and neck cancer. Ann Surg Oncol.

[35] Lu J, Ye X, Fan F, Xia L, Bhattacharya R, Bellister S, Tozzi F, Sceusi E, Zhou Y, Tachibana I, Maru DM, Hawke DH, Rak J, Mani SA, Zweidler-McKay P, Ellis LM (2013). Endothelial cells promote the colorectal cancer stem cell phenotype through a soluble form of Jagged-1. Cancer Cell.

[36] Chen HH, Chakravarty KD, Wang JY, Changchien CR, Tang R (2010). Pathological examination of 12 regional lymph nodes and long-term survival in stages I-III colon cancer patients: an analysis of 2,056 consecutive patients in two branches of same institution. Int J Color Dis.

